# Systemic Corticosteroids and Early Administration of Antiviral Agents for Pneumonia with Acute Wheezing due to Influenza A(H1N1)pdm09 in Japan

**DOI:** 10.1371/journal.pone.0032280

**Published:** 2012-02-29

**Authors:** Koichiro Kudo, Jin Takasaki, Toshie Manabe, Hideko Uryu, Ritsuko Yamada, Emi Kuroda, Nobuyuki Kobayashi, Takeji Matsushita

**Affiliations:** National Center for Global Health and Medicine, Tokyo, Japan; University of Calgary & ProvLab Alberta, Canada

## Abstract

**Background:**

Pneumonia patients with wheezing due to influenza A(H1N1)pdm09 were frequently treated with systemic corticosteroids in Japan although systemic corticosteroid for critically ill patients with pneumonia caused by influenza A(H1N1)pdm09 has been controversial. Applicability of systemic corticosteroid treatment needs to be evaluated.

**Methods/Principal Findings:**

We retrospectively reviewed 89 subjects who were diagnosed with influenza A(H1N1)pdm09 and admitted to a national hospital, Tokyo during the pandemic period. The median age of subjects (45 males) was 8 years (range, 0–71). All subjects were treated with antiviral agents and the median time from symptom onset to initiation of antiviral agents was 2 days (range, 0–7). Subjects were classified into four groups: upper respiratory tract infection, wheezing illness, pneumonia with wheezing, and pneumonia without wheezing. The characteristics of each group was evaluated. A history of asthma was found more frequently in the wheezing illness (55.6%) and pneumonia with wheezing (43.3%) groups than in the other two groups (p = 0.017). Corticosteroid treatment was assessed among subjects with pneumonia. Oxygen saturation was lower in subjects receiving corticosteroids (steroid group) than in subjects not receiving corticosteroids (no-steroid group) (p<0.001). The steroid group required greater oxygen supply than the no-steroid group (p<0.001). No significant difference was found by the Kaplan-Meier method between the steroid and the no-steroid groups in hours to fever alleviation from the initiation of antiviral agents and hospitalization days. In logistic regression analysis, wheezing, pneumonia and oxygen saturation were independent factors associated with using systemic corticosteroids.

**Conclusion:**

Patients with wheezing and a history of asthma were frequently found in the study subjects. Systemic corticosteroids together with early administration of antiviral agents to pneumonia with wheezing and possibly without wheezing did not result in negative clinical outcomes and may prevent progression to severe pneumonia in this study population.

## Introduction

Although systemic corticosteroid treatment for severe pneumonia due to influenza A(H1N1)pdm09 has been controversial [1], [2], [3], systemic corticosteroid treatment in pneumonia patients especially presenting with acute wheezing induced by influenza A(H1N1)pdm09 was frequently administered at the early stage of their illness in hospitals in Japan during pandemic period. Wheezing is the end result of a narrowing of the intrathoracic airways and a limitation of expiratory air flow and is caused by many illnesses. Asthma and bronchiolitis were the main illnesses which caused wheezing in influenza A(H1N1)pdm09 virus infection [4], [5], [6], [7], [8]. Acute exacerbation of asthma is usually diagnosed in patients with wheezing and a history of asthma. It is treated with anti-asthma agents as well as systematic corticosteroids depending on the disease severity following the asthma treatment guidelines [9], [10], [11]. On the other hand, a previous study in preschool children with acute virus-induced wheezing indicated that systemic corticosteroid treatment was not superior to placebo [12]. Also, a study in infants with bronchiolitis concluded that treatment with systemic corticosteroid did not significantly affect hospitalization [13]. It has been physicians' questions whether pneumonia patients presenting with wheezing need to be treated with systemic corticosteroid during the pandemic period.

The aim of the present study was to evaluate if systemic corticosteroid treatment is suitable for hospitalized pneumonia patients with acute wheezing caused by influenza A(H1N1)pdm09.

## Materials and Methods

### Study design

**Figure 1:**
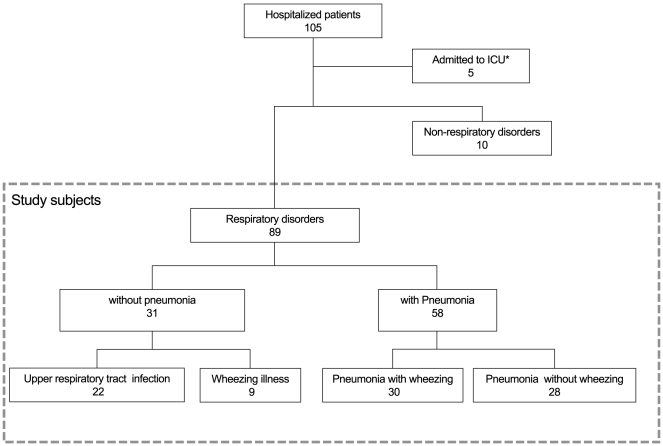
Study population. A total of 104 patients were diagnosed with pandemic influenza A(H1N1)pdm09. Five patients (one of whom died) were admitted to the ICU and were excluded from the study. The remaining 99 patients were the study subjects. Among them, 89 subjects presented with respiratory disorders and 10 presented with symptoms other than respiratory disorders, including encephalopathy. The subjects with respiratory disorders were classified into the following four groups: upper respiratory tract infection (n = 22), wheezing illness (n = 9), pneumonia with wheezing (n = 30), and pneumonia without wheezing (n = 28). The total number of subjects with pneumonia was 58.

We retrospectively reviewed the clinical data, chest radiologic and laboratory findings of all hospitalized patients diagnosed with pandemic influenza A(H1N1)pdm09, admitted between August 2009 and March 2010 to the National Center for Global Health and Medicine (NCGM), which is a tertiary care hospital in Tokyo, Japan. Influenza A(H1N1)pdm09 infection was diagnosed according to case definitions developed by the World Health Organization [14]. Respiratory tract specimens of patients were either tested positive for the influenza A(H1N1)pdm09 virus by real-time reverse-transcriptase-polymerase-chain-reaction (RT-PCR) or tested positive for influenza A virus by ImunoAce Flu® (TAUNS Laboratories, Inc.) or Espline® (Fujirebio Inc.), rapid diagnosis tests using an immunochromatography assay, which are approved by the Ministry of Health, Welfare, and Labour, Japan. Among all hospitalized patients, subjects who presented with respiratory disorders were eligible as study subjects and classified into four groups based on their respiratory disorders: upper respiratory tract infection, wheezing illness, pneumonia with wheezing, and pneumonia without wheezing (Figure 1). The four groups were compared and evaluated in terms of the relationships among clinical conditions, clinical time course, and treatments. The clinical effects for systemic corticosteroids treatment were evaluated among the subjects with pneumonia. Also, clinical factors which leaded to prescribe systemic corticosteroids were assessed among the study subjects. Systemic corticosteroid was administered based on the treatment for acute exacerbation of asthma in the asthma guidelines [9], [10], [11]. Wheezing was defined as a continuous high pitched sound emitting from the chest during expiration on auscultation. Pneumonia was diagnosed on the basis of infiltrative shadows on chest radiograph.

### Statistical analysis

Subjects' background data and clinical laboratory values were summarized and compared among groups of respiratory disorders as well as between those who did (steroid group) and did not (no-steroid group) receive systemic corticosteroid treatment. The Mann-Whitney test and Kruskal-Wallis test were used for continuous variables, and the Chi-square test and Fisher's exact test were used for categorical variables. Survival curves on the numbers of hours to fever alleviation from initiation of administration of antiviral agents and the duration of hospitalization in the steroid and the no-steroid groups were analyzed by the Kaplan-Meier method and comparisons were made using the log-rank test. For the evaluation of independent factors for using systemic corticosteroid treatment, a step-wise selection method was used to select significant factors if p<0.1 in the univariate analysis for a logistic regression analysis. Data analyses were conducted using SPSS statistics ver.19 (IBM, Armonk, NY, USA). For all analyses, significance levels were two tailed, and a p value of <0.05 was considered significant.

### Ethics statement

The study was approved by the Institutional Review Board of the NCGM. Informed consent was waived by the Board for this retrospective study, with the study notification to public being made by posters. Investigators kept the datasets in password-protected systems and presented data with the anonymity of study patients retained.

## Results

### Characteristics of the study subjects

During the study period, a total 104 patients were diagnosed with influenza A(H1N1)pdm09 and admitted to the NCGM. Among them, 89 (85.6%) patients who presented with respiratory disorders were eligible as study subjects (Figure 1). Some subjects were admitted with reasons other than respiratory disorders including encephalopathy, dehydration, and abdominal symptoms due to influenza infection.

The number of subjects in each category of respiratory disorders was as follows: upper respiratory tract infection (n = 22, 24.7%); wheezing illness (n = 9, 10.1%); pneumonia with wheezing (n = 30, 33.7%); and pneumonia without wheezing (n = 28, 31.5%). Of all 89 subjects, the number of subjects with pneumonia was 58 (65.2%).

The characteristics of subjects according to respiratory disorders are shown in Table 1. The median age of study subjects (45 male) was 8 years (range, 0–71), and 80 subjects (89.9%) were aged less than 15 years. More subjects with wheezing illness (55.6%) or pneumonia with wheezing (43.3%) had a history of asthma than did those with upper respiratory tract infection (13.6%) or pneumonia without wheezing (17.9%), and there were significant differences among the groups (p = 0.017). The median oxygen saturation (SpO_2_) in room air on admission in subjects with wheezing illness (91.0%) or pneumonia with wheezing (90.0%) were lower than those in subjects with upper respiratory tract infection (96.5%) or pneumonia without wheezing (93.0%), and there were significant differences among the groups (p<0.001). Bacterial co-infection was detected in throat swabs and/or sputum in 44.9% of subjects, but there was no significant difference among the groups. In terms of laboratory findings, including total serum Immunoglobulin E level, there were no significant differences among the groups.

**Table 1:** Background and clinical characteristics of study subjects.

	Upper respiratory tract infection	Wheezing* illness	Pneumonia† with wheezing	Pneumonia without wheezing	Total	P value
**Number of patients (%)**	22 (24.7)	9 (10.1)	30 (33.7)	28 (31.5)	89 (100.0)	0.007
**Gender, male (%)**	10 (45.5)	9 (100.0)	17 (56.7)	9 (32.1)	45 (50.6)	0.004
**Age-yr.**						0.143
<15	18 (81.8)	8 (88.9)	30 (100.0)	24 (85.7)	80 (89.9)	
≥15	4 (18.2)	1 (11.1)	0 (0.0)	4 (14.3)	9 (10.1)	
**Vaccination**						
Seasonal influenza vaccine of 2009–2011	3 (13.6)	2 (22.2)	7 (23.3)	5 (17.9)	17 (19.1)	0.956
Influenza A(H1N1) pdm09 vaccine	0 (0.0)	5 (27.8)	3 (16.7)	2 (33.3)	10 (18.5)	0.145
**Comorbidity**						
Asthma‡	3 (13.6)	5 (55.6)	13 (43.3)	5 (17.9)	26 (29.2)	0.017
Others§	1 (4.5)	1 (11.1)	0 (0.0)	1 (3.6)	3 (3.4)	0.236
**Family asthma history**	4 (18.2)	3 (33.3)	13 (43.3)	5 (17.9)	25 (28.1)	0.107
**Physical findings**						
Body temperature °C, median (range)	38.5 (35.9–40.4)	38.6 (37.2–38.8)	38.6 (36.5–40.3)	38.6 (36.2–40.2)	38.6 (35.9–40.4)	0.729
SpO_2_ ¶ -%, median (tange)	96.5 (87–98)	91.0 (86–97)	90.0 (82–97)	93.0 (74–98)	92.0 (74–98)	<0.001
Co-infection∥ - No. (%)	6 (27.3)	7 (77.8)	14 (46.7)	13 (46.4)	40 (44.9)	0.081
**Laboratory findings – median (range)**						
WBC (10^3^/µL)	6730 (3260–13980)	15810 (6100–13450)	8000 (2790–16280)	6820 (900–15580)	7740 (900–16280)	0.056
Hemoglobin (g/dL)	13.3 (10.2–16.8)	13.2 (12.0–17.5)	13.4 (4.9–14.9)	13.4 (10.7–15.6)	13.4 (4.9–17.5)	0.911
Platelet (10^3^/µL)	20.3 (8–39)	26.6 (17–44)	23.8 (14–193)	23.2 (12–135)	22.9 (8–193)	0.103
LDH (U/L)	240.5 (168–407)	287.0 (239–397)	270.5 (218–418)	264.5 (183–438)	265.0 (168–438)	0.057
ALP (U/L)	513.5 (7–1173)	748 (240–1091)	620 (449–1008)	603 (123–756)	614.0 (7–1173)	0.224
AST (U/L)	28.0 (16–79)	31.0 (25–50)	29.0 (19–45)	27.0 (21–100)	29.0 (16–100)	0.235
ALT (U/L)	15.0 (8–33)	18.0 (14–33)	13.5 (10–34)	14.5 (8–70)	15.0 (8–70)	0.016
Creatinine (mg/dL)	0.44 (0.22–1.01)	0.3 (0.21–1.03)	0.35 (0.18–0.91)	0.41 (0.26–2.69)	0.40 (0.18–2.69)	0.087
Sodium (mEq/L)	135.0 (129–141)	136 (133–138)	135 (130–141)	135.5 (126–140)	135.0 (126–141)	0.364
Potassium (mEq/L)	3.9 (3.4–5.2)	4.1 (3.5–5.0)	4.0 (3.4–4.6)	4.0 83.3–4.4)	4.0 (3.3–5.2)	0.698
CRP (mg/dL)	0.91 (0–11)	0.83 (0.19–2.06)	1.91 (0.05–9.23)	1.0 (0.07–10.41)	1.17 0.0–11.04)	0.271
Total serum IgE (U/mL)	101.0 (21–6691)	74.0 (3–382)	473.5 (1–9179)	283.0 (25–3440)	243.0 (1–9179)	0.164

* Wheezing was defined as a continuous high pitched sound emitting from the chest during expiration on auscultation.

† Pneumonia was diagnosed on the basis of infiltrative shadows on chest radiograph.

‡ Asthma includes active asthma and inactive asthma.

§ Other comorbidities include smoking , alcoholism, diabetis meritis, chronic heart diseases , obesity.

¶ SpO_2_: oxygen saturation measured by pulse oximetry in room air.

∥ Pathogenic bacteria co-infection was detected by throat swabs and/or sputum.

Definition of abbreviations: WBC, white blood cell count; LDH, lactate dehydrogenase; ALP, alkaline phosphatase; AST, aspartate amino transferase; ALT, alanine aminotransferase; CRP, C-reactive protein; IgE, Immunoglobulin E.

### Treatment and clinical time course of study subjects

The treatments and clinical time courses of study subjects in each classified group of respiratory disorders during hospitalization are shown in Table 2.

**Table 2:** Treatment and clinical time course of study subjects.

	Upper respiratory tract infection	Wheezing* illness	Pneumonia† ^¶^ with wheezing	Pneumonia without wheezing	Total	P value
**Number of subjects (%)**	22 (24.7)	9 (10.1)	30 (33.7)	28 (31.5)	89 (100.0)	0.007
**Treatments**						
Time to initiation of antiviral agents from symptom onset - median days (range)	1.8 (1–3)	1.7 (1–3)	2.4 (1–5)	1.6 (1–7)	1.9 (1–7)	0.054
**Antiviral agents**						0.006
Oseltamivir	12 (54.5)	7 (77.8)	22 (73.3)	12 (42.9)	53 (59.6)	
Zanamivir	9 (40.9)	2 (22.2)	7 (23.3)	13 (46.4)	31 (34.8)	
Both oseltamivir and zanamivir‡	1 (4.5)	0 (0.0)	1 (3.3)	3 (10.7)	5 (5.6)	
**Systemic corticosteroid treatment** §	4 (18.2)	7 (77.8)	28 (93.3)	18 (64.3)	57 (64.0)	<0.001
Time to initiation of systemic corticosteroids from symptom onset - median days (range)	2.0 (2–2)	2.0 (1–5)	2.4 (1–6)	1.8 (1–5)	2.1 (1–6)	0.134
Duration of systemic corticosteroid treatment- median days (range)	5.0 (4–6)	3.3 (2–6)	5.8 (3–9)	4.4 (2–8)	5.2 (2–9)	<0.001
Anti-asthma agents other than corticosteroid¶	6 (27.3)	9 (100.0)	30 (100.0)	21 (75.0)	66 (74.2)	<0.001
Oxygen supply∥	7 (31.8)	5 (55.6)	19 (63.3)	19 (67.9)	50 (56.2)	0.002
Antibiotics**	8 (36.4)	8 (88.9)	28 (93.3)	19 (67.9)	63 (70.8)	<0.001
**Clinical time course**						
Time to fever alleviation* - hours, median (range)	35.4(11–120)	44.0 (14–116)	32.0 (12–150)	37.3 (9–168)	35.0 (9–168)	0.967
Length of Hospitalization, days, median (range)	4.9 (2–9)	6.8 (3–10)	8.4 (6–14)	7.6 (3–14)	7.5 (2–14)	<0.001

* Wheezing was defined as a continuous high pitched sound emitting from the chest during expiration on auscultation.

† Pneumonia was diagnosed on the basis of infiltrative shadows on chest radiograph.

‡ Antiviral medication was switched oseltamivir to zanamivire and vice versa.

§ The dose of corticosteroid was equivalent to methylprednisolone 1.0–1.5 mg/body weight (kg)/time, 2–4 times/day, in subjects under 15 years of age, and 40–80 mg/time, 2–4 times/day in those over 15 years of age.

¶ At least one medication of shortacting β2-agonist, longacting β2-agonist, inhaled isoproterenol, inhaled disodium cromoglycate, aminophylline, and leukotriene receptor antagonists.

∥ Oxygen was administered using a nasal cannula or face mask.

** Antibiotics.

All subjects were treated with antiviral agents, either oseltamivir or zanamivir. In some subjects antiviral medication was switched from oseltamivir to zanamivir or vice versa. The regular dose of oseltamivir was 150 mg/day for 5 days in adults, and 4 mg/kg/day for 5 days in pediatric patients. The regular dose of zanamivir was 20 mg/day for 5 days. The median number of days from symptom onset to initiation of administration of antiviral agents was 1.9 (range, 1–7), and the length to antiviral treatment from the symptom onset in the pneumonia with wheezing group tended to be longer (2.4 days; range, 1–5) (p = 0.054).

Systemic corticosteroid was used in 93.3% of pneumonia with wheezing subjects, 77.8% of wheezing illness subjects, and 64.3% of pneumonia without wheezing subjects (p<0.001). The dosage of corticosteroids was equivalent to methylprednisolone 1.0–1.5 mg/body weight (kg)/time, 2–4 times/day, in subjects under 15 years of age, and 40–80 mg/time, 2–4 times/day in those over 15 years of age. The median number of days from symptom onset to initiation of administration of systemic corticosteroids was 2.1 (range, 1–6), The median duration of systemic corticosteroid treatment was 5.2 days (range, 2–9).

Treatment with anti-asthmatic agents other than corticosteroids were included in drug regimens for asthmatic episodes, cough and sputum using at least one of the following: short-acting β_2_-agonist, long-acting β_2_-agonist, inhaled isoproterenol, inhaled disodium cromoglycate, aminophylline, and leukotriene receptor antagonist. All subjects in wheezing illness and pneumonia with wheezing groups received anti-asthma treatments; also, 27.3% of those with respiratory tract infections and 75.0% of those with pneumonia without wheezing had at least one administration with anti-asthma agent (p<0.001).

Oxygen was administered using a nasal cannula or face mask to 56.2% of subjects with respiratory disorders, but no subjects required mechanical ventilation.

The time to fever alleviation from the initiation of administration of antiviral agents was not significantly different among the groups (p = 0.967). There was a longer duration of hospitalization in the pneumonia groups with and without wheezing compared with the other two groups, and there was significant difference among the groups (p<0.001).

### Evaluation of systemic corticosteroid treatment among subjects with pneumonia

Systemic corticosteroid treatment was evaluated in subjects with pneumonia (n = 58) and compared between subjects in the steroid group and in the no-steroid group (Table 3).

**Table 3:** Clinical presentation of subjects with pneumonia according to systemic corticosteroid treatment.

	Steroid group*	No-steroid group*	Total	P value
**Number of subjects No. (%)**	46 (100)	12 (100)	58 (100)	
**Symptoms and signs on admission**				
Wheezing† -No. (%)	27 (58.7)	1 (8.3)	28 (48.3)	0.002
Co-infection‡-No. (%)	24 (52.2)	3 (25.0)	27 (46.6)	0.093
Body temperature -°C, median (range)	38.6 (36.5–40.3)	38.2 (36.2–40.2)	38.6 (36.2–40.3)	0.261
**Laboratory findings on admission, median (range)**				
SpO_2_ § (%)	90.0 (74–97)	95.6 (91–98)	91.0 (74–98)	<0.001
WBC (10^3^/µL)	8200 (2790–16280)	6385.0 (900–13280)	7715.0 (900–16280)	0.024
LDH (U/L)	270 (201–418)	255.5 (183–438)	267.5 (183–438)	0.687
CRP (mg/dL)	1.16 (0.05–9.23)	2.69 (0.07–10.41)	1.22 (0.05–10.41)	0.154
Sodium (mEq/L)	135.1 (130–141)	134.8 (126–139)	135 (126–141)	0.734
Potassium (mEq/L)	3.96 (3.3–4.6)	3.39 (3.3–4.5)	3.95 (3.3–4.6)	0.438
**Treatment -No. (%)**				
Days to administration of antiviral agents¶	2.0 (1–5)	1.8 (1–7)	2.0 (1–7)	0.589
Anti-asthma treatments∥	45 (97.8)	6 (50.0)	51 (87.9)	<0.001
Antibiotic agents	41 (89.1)	6 (50.0)	47 (81.0)	0.006
Oxygen supply**	45 (97.8)	1 (8.3)	46 (79.3)	<0.001
**Clinical outcomes, median (range)**				
Hours to alleviation of fever after admission††	36.0 (9–150)	35.5 (9–168)	35.5 (9–168)	0.611
Hospitalization days	8.2 (5–14)	7.7(3–14)	8.1 (3–14)	0.607

N = 58.

* No-steroid and steroid group denote group of subjects who were not treated and treated with systematic corticosteroids.

† Wheezing was defined as a continuous high pitched sound emitting from the chest during expiration on auscultation.

‡ Pathogenic bacteria co-infection was detected by throat swabs and/or sputum.

§ SpO_2_: oxygen saturation measured by pulse oximetry in room air.

¶ The number of days from symptom onset to the initiation of administration of antiviral agent either oseltamivir or zanamivir.

∥ At least one medication of short-acting β2-agonist, long-acting β2-agonist, inhaled isoproterenol, inhaled disodium cromoglycate, aminophylline, and leukotriene receptor antagonists.

** Oxygen was administered using a nasal cannula or face mask.

†† The time (hours) to alleviation of fever to less than 37°C after the administration of antiviral agents.

Definition of abbreviation: WBC, white blood cell count; LDH, lactate dehydrogenase; CRP, C-reactive protein.

Wheezing was presented 58.7% in the steroid group and there was a significant difference between the groups (p = 0.002). SpO_2_ in the steroid group was lower than that in the no-steroid group (SpO2, 90.0% vs. 95.6, respectively; p<0.001) and the steroid group required more oxygen supply than the no-steroid group (97.8% vs. 8.3%, respectively; p<0.001). Anti-asthma treatment was applied to 97.8% of the steroid group and 50.0% of the no-steroid group (p<0.001). Although bacterial co-infection was found in 52.2% of the steroid group and 25.0% of the no-steroid group at the time of admission (p = 0.093), antibiotics were administered to both the steroid and the no-steroid groups (89.1% vs. 50.0%, respectively; p = 0.006). There were no significant differences in terms of time to fever alleviation (<37°C) after administration of antiviral agents and in the duration of hospitalization between the groups (p = 0.611 and 0.599, respectively).

**Figure 2:**
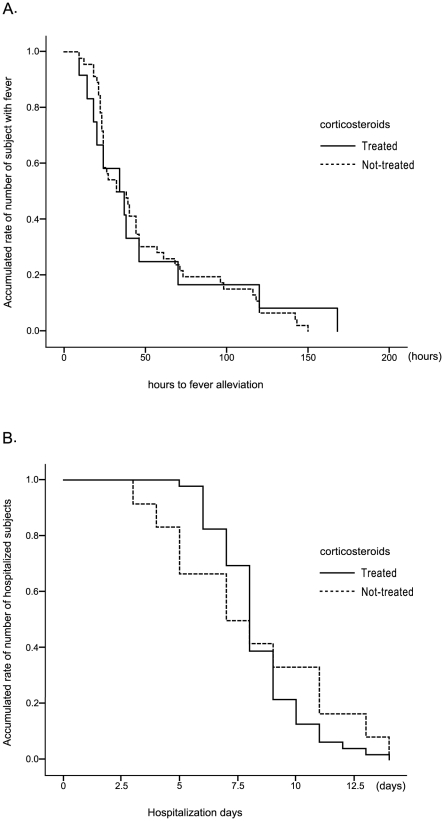
Systemic corticosteroids treatment in the relation0 to clinical time course assessed by Kaplan-Meier methods. Kaplan-Meier curves of the number of hours to fever alleviation (A) and hospitalization days (B) according to systemic corticosteroid treatment among subjects with viral pneumonia in steroid (n = 46) and non-steroid (n = 12) groups There were no significant differences between the groups in terms of either hours to fever alleviation (log rank test, p = 0.835) or hospitalization days (log rank test, p = 0.626).

Clinical time course were assessed by the Kaplan-Meier method on time to fever alleviation from the initiation of administration of antiviral agents and duration of hospitalization in subjects with pneumonia (n = 58) and compared between the steroid and the no-steroid groups using the log-rank test (Figure 2). There were no significant differences between the groups in both time to fever alleviation (p = 0.835) and the duration of hospitalization (p = 0.626).

### Clinical factors for using systemic corticosteroids treatment among the study subjects

A multiple logistic regression analysis using baseline factors was conducted for subjects with respiratory disorders (n = 89). Wheezing, pneumonia and SpO_2_ on admission were independent clinical factors associated with using systemic corticosteroids treatment (Table 4).

**Table 4:** Clinical factors for using systemic corticosteroids treatment among the study subjects by multiple logistic regression analysis.

Parameter	Regression coefficient	Standard error	P value	Odds ratio	95% confidence interval
Intercept	20.444	8.927			
Wheezing*	2.401	0.841	0.004	11.03	2.12–57.33
Pneumonia†	1.298	0.618	0.036	3.66	1.09–12.30
SpO2‡	−0.229	0.094	0.015	0.80	0.66–0.96

n = 89.

* Wheezing was defined as a continuous high pitched sound emitting from the chest during expiration on auscultation.

† Pneumonia was diagnosed on the basis of infiltrative shadows on chest radiograph.

‡ SpO_2_: oxygen saturation measured by pulse oximetry in room air on admission.

## Discussion

Our evaluation of hospitalized patients with pneumonia caused by influenza A(H1N1)pdm09, who were mostly young and presenting with wheezing, revealed that early systemic corticosteroid treatment did not result in negative clinical outcomes if patients were treated with antiviral agents during the early stage of illness.

Although asthma is not the only illness that causes wheezing, asthma is a risk comorbidity for influenza A(H1N1)pdm09 [8]. In the present study, wheezing was observed in 43.8% of all subjects, including in subjects who presented with pneumonia (Table 1). Systemic corticosteroid treatment is recommended in the asthma guidelines for treating acute exacerbation of asthma which requires hospitalization [9], [10], [11], but its use remains uncertain for asthma-exacerbated patients with pneumonia due to influenza A(H1N1)pdm09 [1], [2], [3].

The healthcare seeking behavior for people in Japan is customarily early especially for acute diseases including pandemic influenza and a median days to the initiation of treatment with antiviral agents from the symptom onset was 1.9 days (range, 1–7) in the study subjects (Table 2). The previous study in Mexico reported that the earlier administration of antiviral agent reduced severity of pneumonia, occurrence of pneumonia, and the duration of hospitalization [15]. In the present study, the study subjects were not admitted in the ICU, did not require mechanical ventilation support, and the median of duration of hospitalization was 7 days (range, 2–14) (Table 1). These results indicated that the study subjects, who were mostly young and were initiated the treatment with antiviral agents earlier, did not progress to be critical.

Most subjects presented with respiratory disorders, including upper respiratory tract infection, wheezing illness, and pneumonia with or without wheezing. Wheezing is one of the manifestations of asthma and a previous report indicated that wheezing is associated with influenza mortality [16]. In the present study, total 43.8% of subjects presented wheezing and 33.7% presented both pneumonia and wheezing. Also, asthma history was more frequent among subjects with wheezing illness (55.6%) or pneumonia with wheezing (43.3%) than among the other subjects in upper respiratory infection (13.6%) and pneumonia without wheezing (17.9%) (Table 1). The results indicated that the occurrence of exacerbation of asthma might have been increased by influenza A(H1N1)pdm09 in this study population; however, exacerbation of asthma did not became critical. Systemic corticosteroid was mainly used for patients with wheezing either with pneumonia or without pneumonia. The presentation of wheezing can lead to a diagnosis of asthma if the patients have a history of asthma. Also, wheezing could also indicate a first episode of asthma attack or bronchiolitis, which are difficult to distinguish on the basis of wheezing alone. If a patient has asthma acute exacerbation or a first episode of asthma attack, not using systemic corticosteroids can increase disease severity and mortality [9], [10], [11]. Therefore, systemic corticosteroid treatment should be a consideration for clinical management of patients with wheezing despite the presence of viral pneumonia and/or bronchiolitis. Also, anti-asthmatic agents other than systemic corticosteroids were administered to all subjects with wheezing (Table 2). Treatments with anti-asthmatic agents together with corticosteroids need to be included as well as antibiotic agents in case of bacterial co-infection.

Respiratory condition, as reflected by SpO_2_, was more severe in subjects of the steroid group than in the non-steroid group (Table 3). Also, oxygen was more supplied to the steroid group. Those results described that respiratory condition was more severe in the steroid group; however, systemic corticosteroid treatment has no influence to hours to fever alleviation after the initiation of treatment with antiviral agents and the duration of hospitalization (Table 3). In terms of assessment of clinical time course, the Kaplan-Meier curves for hours to fever alleviation from the initiation of antiviral agents and hospitalization days were not significantly different between the steroid and the non-steroid groups. (Figure 2). Systemic corticosteroids were administered for the most of subjects with wheezing (Table 2). These results suggests that systemic corticosteroid treatment for viral pneumonia with wheezing may not have negative effects to clinical time course.

Multiple logistic regression analysis among subjects with respiratory disorders evaluated that wheezing, pneumonia, and SpO_2_ were independent factors associated with using systemic corticosteroid treatment (Table 4). The results indicated that these were factors that could motivate physicians to start systemic corticosteroid treatment at the study site. It also indicated that wheezing was not the only factor for using systemic corticosteroid treatment but also pneumonia and low level of respiratory condition which were reflected by SpO_2_. In the present study, systemic corticosteroid treatment did not produce negative outcomes, even in patients with pneumonia and might be in patients with bronchiolitis. The results showed that the systemic corticosteroid treatment in the early stage of illness together with antiviral agents might work to reduce the time of critical conditions and to prevent disease progression to severe pneumonia among patients who were administered antiviral agents during the early stage of illness when their pneumonia were not so severe (Table 2).

Although secondary bacterial infection was reported as a negative outcome of systemic corticosteroid treatment in the severe ill patients with influenza H1N1pdm09 [2], in the present study, no significant effects of systemic corticosteroid treatment against bacterial co-infection were observed. It might be resulted from the antibiotics treatment from the hospital admission (Table 2, 3) and can be explained by the short duration of hospitalization of study subjects. (Table 2, 3).

Limitations of the present retrospective study are that the influenza HN1 2009 virus was confirmed in the limited number of subjects by RT-PCR and patients strongly considered to have 2009 influenza A/H1N1 virus infection were included. During the study period, influenza A(H1N1)pdm09 virus was the dominant influenza virus in Japan according to the Infectious Agent Surveillance Report in Japan [17]. Subjects who were identified as having influenza A virus infection were strongly considered to have influenza A(H1N1)pdm09 virus infection, so physicians diagnosed those patients as having influenza A(H1N1)pdm09 infection. Also, most of the study subjects were pediatric patients and the age distribution of the study subjects was representative of that for influenza A(H1N1)pdm09 in Japan [17], [18]. The number of subjects in divided four groups according to the respiratory conditions were not equal as well as the small number of subjects without steroids treatment due to the retrospective study in a single hospital. Therefore, the further prospective study in patients with a variety of ages with large population is needed.

In conclusions, systemic corticosteroid treatment together with early administration of antiviral agents did not result in negative clinical outcomes in patients with influenza viral pneumonia with wheezing and without wheezing in the present study. The findings suggest that influenza pneumonia patients with wheezing and potentially without wheezing could be treated by systemic corticosteroids and early administration of antiviral agents if the severity of disease is before critical condition.

## References

[1] Brun-Buisson C, Richard JC, Mercat A, Tiebaut AC, Brochard L, for the REVA-SRLF A/H1N1 v 2009 Registry Group (2011). Early Corticosteroids in Severe Influenza A/H1N1 Pneumonia and Acute Respiratory Distress Syndrome.. Am J Respir Care Med.

[2] Kim SH, Hong SB, Yun SC, Choi WI, Ahn JJ (2011). Corticosteroid Treatment in Critically Ill Patients with Pandemic Influenza A/H1N1 2009 Infection: Analytic Strategy Using Propensity Scores.. Am J Respir Care Med.

[3] Hong-Ryang K, Jae-Ho L, Kyung-Yil L, Jung-Woo R, You-Sook Y (2011). Early corticosteroid treatment for severe pneumonia caused by 2009 H1N1 influenza virus.. Critical Care.

[4] Perez-Padilla R, de la Rosa-Zamboni D, Ponce de Leon S, Hernandez M, Quiñones-Falconi F (2009). Pneumonia and respiratory failure from swine-origin influenza A (H1N1) in Mexico.. N Engl J Med.

[5] Jaian S, Kamimoto L, Bramley AM, Schmitz AM, Benoit SR (2009). Hospitalized patients with 2009 H1N1 influenza in the United States, April–June 2009.. N Engl J Med.

[6] Webb SA, Aubron C, Bailey M, Bellomo R, The ANZIC Influenza Investigators (2009). Critical care services and 2009 H1N1 influenza in Australia and New Zealand.. N Engl J Med.

[7] Louie JK, Acosta M, Jean C, Gavali S, Schechter R (2009). Factors Associated With Death or Hospitalization Due to Pandemic 2009 Influenza A(H1N1) Infection in California.. JAMA.

[8] Bautista E, Chotpitayasunondh T, Gao Z, Harper SA, Shaw M, Writing Committee of the WHO Consultation on Clinical Aspects of Pandemic (H1N1) 2009 Influenza (2010). Clinical aspects of Pandemic 2009 Influenza A (H1N1) virus infection.. N Engl J Med.

[9] The Global Initiative for Asthma(GINA) (2010). Global Strategy for Asthma Management and Prevention.. http://www.ginasthma.org/pdf/GINA_Report_2010.pdf.

[10] National Asthma Education and Prevention Program (2007). Guidelines for the Diagnosis and Management of Asthma, Expert Panel ReportIII.

[11] Japanese Society of Allergoology (2009). Asthma Prevention and Management Guidelines 2009, Japan.

[12] Panickar J, Lakhanpaul M, Lambert PC, Kenia P, Stephenson T (2009). Oral Prednosolone for Preschool Children with Acute Virus-Induced Wheezing.. N Engl J Med.

[13] Corneli H, Zorc JJ, Mahjan P, Shaw KN, Holubkov R (2007). A Multicenter, Randomized, Controlled Trial of Dexamethasone for Bronchiolitis.. N Engl J Med.

[14] World Health Organization (2010). WHO guidelines for pharmacological management of pandemic (H1N1) 2009 influenza and other influenza viruses.. http://www.who.int/csr/resources/publications/swineflu/h1n1_guidelines_pharmaceutical_mngt.pdf.

[15] Higuera Iglesias AL, Kudo K, Manabe T, Corcho Berdugo AE, Baeza AC (2011). Reducing Occurrence and Severity of Pneumonia Due to Pandemic H1N1 2009 by Early Oseltamivir Administration: A Retrospective Study in Mexico.. PLoS ONE.

[16] Riquelme R, Jimenez P, Videla AJ, Lopez H, Chalmers J (2010). Predicting morality in hospitalized patients with 2009 H1N1 influenza pneumonia.. Int J Tuberc Lung Dis.

[17] Infectious Disease Surveillance Center (2009). Pandemic influenza A(H1N1) situation report of Japan, update 27.. http://idsc.nih.go.jp/disease/swine_influenza_e/idsc_e2009/09idsc27e.html.

[18] The Ministry of Health, Labour, and Welfare (2011). The trend of pandemic H1N1 2009: Epidemiological information for medical providers ver. 3.. http://www.mhlw.go.jp/bunya/kenkou/kekkaku-kansenshou04/pdf/100423-01.pdf.

